# Comparative Evaluation of the Capacity of Commercial and Autochthonous *Saccharomyces cerevisiae* Strains to Remove Ochratoxin A from Natural and Synthetic Grape Juices

**DOI:** 10.3390/toxins14070465

**Published:** 2022-07-07

**Authors:** Islem Dammak, Norah Salem Alsaiari, Imene Fhoula, Abdelfattah Amari, Zohra Hamdi, Mnasser Hassouna, Faouzi Ben Rebah, Tahar Mechichi, Salma Lasram

**Affiliations:** 1 Laboratory of Technological Innovation and Food Security LR22-AGR01, Higher School of Food Industries of Tunisia (ESIAT), University of Carthage, Tunis 1003, Tunisia; islem.dammak.a@gmail.com (I.D.); mnasser.hassouna@isbb.rnu.tn (M.H.); 2 Laboratory of Biochemistry and Enzymatic Engineering of Lipases, National Engineering School of Sfax (ENIS), University of Sfax, Sfax 3000, Tunisia; 3 Department of Chemistry, College of Science, Princess Nourah bint Abdulrahman University, P.O. Box 84428, Riyadh 11671, Saudi Arabia; nsalsaiari@pnu.edu.sa; 4 Laboratory of Microorganisms and Actives Biomolecules, Faculty of Sciences of Tunis, University of Tunis El Manar, Tunis 2092, Tunisia; imene.fhoula@fst.utm.tn; 5 Department of Chemical Engineering, College of Engineering, King Khalid University, Abha 61411, Saudi Arabia; abdelfattah.amari@enig.rnu.tn; 6 Research Laboratory of Processes, Energetics, Environment and Electrical Systems, Department of Chemical Engineering & Processes, National School of Engineers, Gabes University, Gabes 6072, Tunisia; 7 Laboratory of Molecular Physiology of Plants, Center of Biotechnology of Borj Cedria (CBBC), BP 901, Hammam-Lif 2050, Tunisia; zohrahamdicbbc@gmail.com (Z.H.); salma.lasram.cbbc@gmail.com (S.L.); 8 Higher Institute of Biotechnology of Sfax, University of Sfax, Sfax 3000, Tunisia; benrebahf@yahoo.fr

**Keywords:** grape juice, ochratoxin A, adsorption, *Saccharomyces cerevisiae*

## Abstract

In this paper, we assessed the ability of two strains of *Saccharomyces cerevisiae*, in viable and dead forms, to remove ochratoxin A (OTA) from an artificially contaminated synthetic grape juice medium (SGM) (10 µg OTA/L) and a naturally contaminated grape juice (6.64 µg OTA/L). The first strain, named Levulin FB, is a commercial yeast used in making wine. The second, named SC5, is an autochthonous strain isolated from table grapes. OTA concentrations in juices before and after their contact with yeast cells were assessed. A significant decrease in OTA level (*p* < 0.05) in the SGM medium and in the natural grape juice was observed after 1 h of adding yeast cells (20 g/L) in viable and heat-treated forms. It was inferred that the dead forms of the two strains were more able to eliminate OTA than their viable forms in both media. This study demonstrates the potential application of an autochthonous yeast for the natural decontamination of grape juice from fungal toxins.

## 1. Introduction

Ochratoxin A (OTA) is a potent toxin generated by many fungal species belonging to *Penicillium* and *Aspergillus* genera. *Penicillium verrucosum*, *Aspergillus ochraceus*, *A. niger aggregate* and *A. carbonarius* are the main OTA-producing species [1]. OTA reveals genotoxic, nephrotoxic, carcinogenic, teratogenic, immunosuppressive and hepatotoxic effects [2]. OTA was firstly mentioned by Zimmerli and Dick [3] in wine and grape juice. The presence of this mycotoxin is a result of grape colonization by ochratoxigenic fungi during pre- and post-harvest periods under favorable environmental conditions. The potential OTA production of fungi species depends largely on the geographical location of vines. In fact, several studies reported that grapes and their derived products in the Mediterranean regions of south Europe and north Africa were the most contaminated by OTA [4,5,6]. Furthermore, Lasram et al. [7] suggested that grape products in the Mediterranean countries were highly contaminated by OTA, and *A. carbonarius* is regarded as the basic ochratoxigenic fungus in wines. Lasram et al. [8] reported in another investigation that the Tunisian grape must contain high levels of OTA, varying between 0.05 and 5.85 µg/L. However, the European Union legislation authorities have limited the OTA to the level of 2 µg/L in grape juice, wine and must [9]. Different methods were applied to reduce the level of OTA and hence prevent the contamination of grapes by the ochratoxigenic fungi in the pre- and post-harvest phases or during the phases before the transformation of grapes [6,10]. However, the contamination frequently persists, and the berries are infected with OTA. For this reason, detoxification of grape products from OTA requires an alternative method. So far, different strategies for the elimination of OTA have included the use of chemical, physical and microbiological procedures [11]. Piotrowska [12] considered that the use of microbiological methods to remove mycotoxin seems to be a better solution compared to the chemical approaches because these methods achieve decontamination while maintaining the nutritional value and palatability of products. In this context, Amézqueta et al. [13] asserted that some fungi, including *Botrytis*, *Aspergillus*, *Penicillium* and *Alternaria* genera, and some bacteria, such as *Bifidobacterium*, *Streptococcus* and *Bacillus,* were able to remove OTA from food products. In addition, several studies confirmed that the most promising decontamination methods include the use of microorganisms, especially lactic acid bacteria and yeasts [14,15]. In fact, the use of yeasts to eliminate OTA from grape juice, wine and must proved to be more powerful [16,17]. Moreover, there is no consensus about the mechanisms of removing OTA. Indeed, Angioni et al. [18] clarified that certain strains of yeast can decrease the rate of OTA in wine via the mechanism of degradation. Meanwhile, several authors underlined that yeasts can remove OTA through the mechanism of adsorption [17,19]. On the other hand, multiple studies reported that heat treatment of yeast cells can enhance their capacity to adsorb OTA, probably owing to the increase in adsorption of cell sites after this operation [20]. Additionally, Ringot et al. [21] considered the yeast biomass as an effective adsorbing tool in view of the presence of specific macromolecules, including β-glucans and mannoproteins. Moreover, some authors assumed that the major cellular components used in the mechanism of OTA binding by yeast cells are polyparietal saccharides (glucan, mannan) [16,22]. From this perspective, the use of *Saccharomyces* yeasts to detoxify derived grape products seems to be a solution referring to the rich composition of the cell walls of polysaccharides and peptidoglycans [23]. Thus, the aim of this study was to assess the ability of the newly isolated yeast strain *S. cerevisiae* SC5 to remove OTA from synthetic and natural grape juices and to compare its capacity (viable and dead cells) to remove OTA to that of the commercial oenological *S. cerevisiae* strain.

## 2. Results

### 2.1. Decontamination of OTA from Synthetic Grape Juice

**Figure 1:**
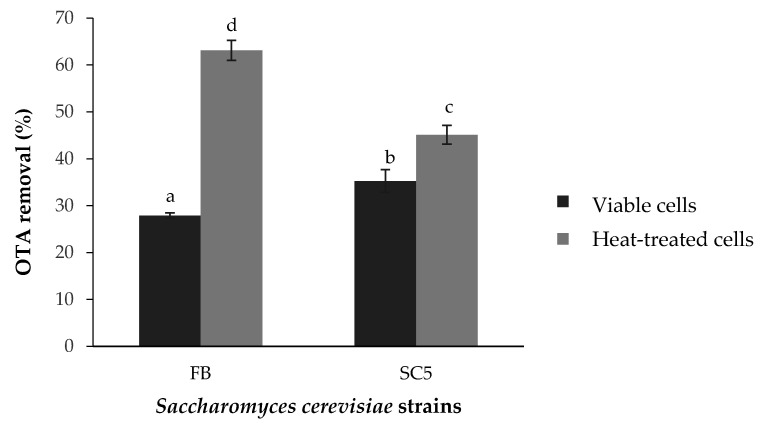
OTA removal percentage in synthetic grape juice. Different letters indicate a significant difference according to Duncan test (*p* < 0.05). Bars represent mean values of three replicates ± SD.

The effects of the inoculation of dead cells obtained by heat treatment and viable cells of *S. cerevisiae* yeasts—SC5 (autochthonous yeast isolated from table grape) and Levulin FB (commercial yeast used in winemaking)—on OTA reduction in synthetic grape juice (SGM medium) artificially contaminated with 10 µg/L of this mycotoxin are shown in Table 1 and Figure 1. Data in Table 1 and Figure 1 are means ± standard deviation (SD) of triplicate assays. Analysis of variance (ANOVA) revealed that both viable and heat-treated cells of both yeasts were able to significantly decrease the concentration of OTA in synthetic medium after 1 h of incubation at 30 °C (*p* < 0.05). In addition, the viability state of yeast cells has a significant effect on the decontamination capacity of OTA. In fact, heat-treated cells displayed an OTA elimination ability that is superior to that of living cells (*p* < 0.05). Results demonstrated that OTA concentrations in SGM medium after the addition of heat-treated cells of Levulin FB and SC5 were 3.63 ± 0.21 and 5.41 ± 0.1 µg/L, respectively (Table 1). The inhibition rate was 63.10 ± 2.15% for Levulin FB and 45.11 ± 1.98% for SC5 (Figure 1). However, living cells of Levulin FB and SC5 reduced OTA concentrations in the medium to 7.11 ± 0.06 and 6.38 ± 0.23 µg/L, respectively (Table 1). These values correspond to inhibition percentages of 27.8 ± 0.56% and 35.27 ± 2.42%, respectively (Figure 1). It seems that autochthonous *S. cerevisiae* is more efficient in detoxifying the OTA medium than the commercial strain Levulin FB (*p* < 0.05) when their cells are inoculated in the viable form.

### 2.2. Decontamination of OTA from Natural Grape Juice

**Figure 2:**
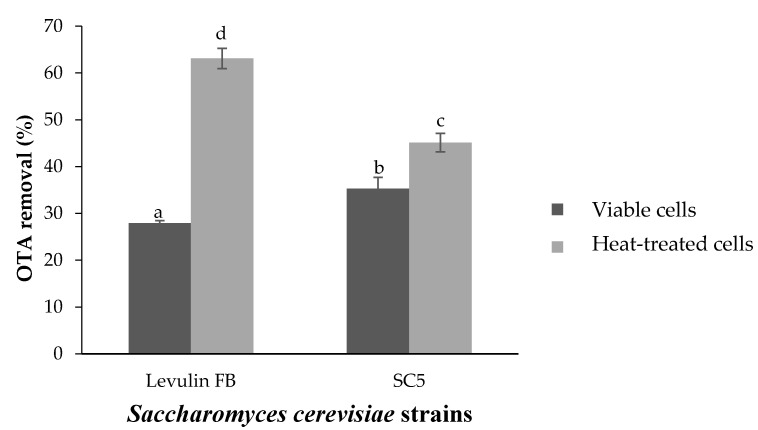
OTA removal percentage in natural grape juice. Different letters indicate a significant difference according to Duncan test (*p* < 0.05). Bars represent mean values of three replicates ± SD.

The natural grape juice was contaminated with 6.64 µg/L of OTA. Results relating to the effect of SC5 and Levulin FB cell inoculation on OTA reduction in the grape juice are depicted in Table 2 and Figure 2. Data in Table 2 and Figure 2 are means ± SD of triplicate assays. Analysis of variance (ANOVA) proved that reductions were all significant compared to the control (*p* < 0.05). The viability of cells had a significant influence on OTA decontamination (*p* < 0.05). Indeed, the addition of heat-treated cells reduced the OTA concentration in the medium more significantly than viable cells (*p* < 0.05). Statistical analysis suggested that heat-treated cells of the commercial *S. cerevisiae* strain (Levulin FB) decreased OTA levels in natural grape juice more significantly than those of the autochthonous strain (SC5). In fact, dead cells of Levulin FB and SC5 reduced OTA levels in the juice to 3.36 ± 0.09 and 4.02 ± 0.03 ng/mL, respectively, corresponding to decontamination rates of 49.77 ± 1.90 and 39.46 ± 0.41%, respectively (Figure 2). On the other side, viable cells of Levulin FB and SC5 decreased OTA concentration in the medium to 5.21 ± 0.11 and 4.46 ± 0.22 µg/L, respectively, yielding percentages of decontamination of 21.59 ± 1.63 and 32.86 ± 0.34%, respectively. Statistical analysis confirmed that viable cells of the newly isolated strain SC5 significantly removed OTA from natural grape juice much better than the commercial strain Levulin FB (*p* < 0.05).

## 3. Discussion

The presence of OTA on agricultural products and fruit juices stands for a serious threat to health. This work highlights the efficiency of yeast cells in eliminating this mycotoxin from natural and synthetic grape juices. The efficiency of *S. cerevisiae* in eliminating mycotoxin was suggested by multiple authors. Within this framework, Bueno et al. [24] and Pizzolitto et al. [25] pointed out that this species of yeast was efficient in removing aflatoxin B1, which is considered the most cancerogenic mycotoxin. Additionally, Bejaoui et al. [26] proved the effectiveness of this species in decontaminating synthetic grape juice from OTA. In this work, two *S. cerevisiae* strains were used for the decontamination of grape juice from OTA. The first is a natural strain isolated from table grape, and the second is an industrial strain used in the winemaking process. These two strains were tested to determine their ability to decontaminate grape juice (natural and synthetic) from OTA. Results demonstrated that both tested strains of *S. cerevisiae* were able to decontaminate natural grape juice (6.64 µg/L of OTA) and the artificially contaminated medium (SGM) (9.81 µg/L of OTA) from OTA. In fact, viable cells of *S. cerevisiae* SC5 removed more OTA from natural and synthetic grape juice than the industrial strain (Levulin FB), with removal percentages of 32.86 ± 0.34% and 35.26 ± 2.42%, respectively. From this perspective, this newly isolated strain could be of interest to the winemaking industry because the strain could be used for the elimination of OTA from wine as well as for reinforcing the process of winemaking after checking its fermentation potential.

Results also indicated that dead yeasts are more efficient than growing yeasts in terms of decontaminating both synthetic and natural grape juice from OTA. This observation agrees with the research reported by Bejaoui et al. [26], which proved that heat-treated cells of *S. cerevisiae* and *S. bayanus* are much more efficient in removing OTA than viable cells. Additionally, Petruzzi et al. [27] asserted that the viability of *Saccharomyces* cells is not a precondition to decontaminating a model wine buffer from OTA. Thus, the mechanism adopted in reducing the OTA concentration in the medium is one of adsorption rather than of catabolism. This mechanism of adsorbing the mycotoxin was also examined with lactic acid bacteria, whose dead cells were able to adsorb zearalenone, α-zearalenol, trichotecenes and aflatoxin [28]. The use of yeast cells as an adsorbing material to eliminate OTA is very advantageous compared to the application of inorganic compounds such as activated carbon, aluminosilicates, bentonites and zeolites, which deteriorate the organoleptic properties of foods and reduce their nutritive value [29]. Notably, El-Sharkawy et al. [30] underlined that the mechanism of adsorption is a promising process, referring to the absence of degradation metabolites which could be even much more toxic than OTA, as has been inferred with zearalenone. This study proved that the mechanism of removing OTA from grape juice is very fast. In fact, heat-treated cells of both tested strains ensured more than a 39% reduction in OTA in both tested media over 1 h of contact. Within the same framework, Shetty et al. [31] demonstrated that the elimination of OTA from grape juice is fast due to its rapid adsorption by the walls of yeast cells.

It can be noticed that the rate of OTA elimination by dead cells of both tested strains from natural grape juice was approximately double that recorded by viable cells. This observation is consistent with previous studies [31,32]. This further confirms that the application of dead cells ensures a very high percentage of mycotoxin binding compared to that exerted by living cells, and the rate of decontamination by the former can be twice as high as that of the latter. According to previous investigations, the adsorption of OTA mycotoxin was achieved by the cell walls because most of the constituents of the cell-like polysaccharides, proteins and lipids are located at the level of the wall. These constituents represent highly accessible sites for the adsorption process. They also display ionic or hydrophobic interactions and hydrogen bonds [33]. In fact, Ringot et al. [22] emphasized that the adsorption of OTA mycotoxin by yeasts is basically ensured by β-glucans and mannoproteins located in cell walls. These two constituents present negative charges against the pH of grape juice, which cause polar and nonpolar interactions with OTA. In this context, numerous studies suggested that β-(1,3 and 1,6)-D-glucans molecules situated in the cell wall of *S. cerevisiae* are the most responsible agents for mycotoxin adsorption [34,35]. The variability in terms of efficiency of invested strains to eliminate OTA from grape juice may be attributed to the different internal composition of the peptidoglycans in cell walls responsible for the adsorption phenomenon [36]. The high efficiency of heat-treated cells in removing OTA from natural and synthetic grape juice could be interpreted as being due to the fact that heat treatment affects peptidoglycans and polysaccharides, triggering the denaturation of proteins and the formation of numerous products following the Maillard reaction. Obtained products can generate multiple adsorption sites and can increase the OTA binding surface area. Heat treatment yields multiple changes in the cell wall of peptidoglycan, such as reduction of its thickness and expansion in the size of its pores [28]. According to previous studies, the ability of yeast to adsorb toxin depends not only on the inoculated yeast products (living cells, heat- or acid-treated cells, or cell wall) but also on the type of mycotoxin (AFB1, OTA, ZEA) [37]. Moreover, several authors showed that the rate of toxin decontamination depends largely on the concentration of yeast as well as the total amount of the cell wall [29,38]. Likewise, Yiannikouris et al. [39] found that the adsorption rate of mycotoxin is influenced by the content of β-D-glucan and its three-dimensional arrangement in the cell wall.

As for the grape juice, which does not require fermentation, *S. cerevisiae* strains which are able to remove OTA and do not rely on cell viability are excellent adsorbents. From this perspective, the application of inactivated biomass of autochthonous strain SC5 for OTA adsorption provides a new promising application in the food industry. In terms of winemaking, it is possible to use viable cells of the newly isolated strain SC5 after checking its fermentation capacity. The ability of this strain to remove OTA is high compared to that of the commercial strain Levulin FB.

## 4. Conclusions

To conclude, both tested yeast strains of *S. cerevisiae*: SC5 (autochthonous yeast isolated from table grape) and Levulin FB (commercial yeast used in winemaking) were able to remove OTA from synthetic and natural grape juices when they were inoculated in viable and dead forms, with an important capacity for dead cells. Therefore, it would be interesting to propose the use of dead cells of the strain SC5 by the food and winemaking industries as an efficient biological treatment to detoxify grape juice, must and wine without negative effects on human health.

## 5. Materials and Methods

### 5.1. Chemicals

A stock standard solution of OTA (Sigma-Aldrich, St. Louis, MO, USA) was prepared by dissolving 1 mg of OTA standard in 1 mL of pure methanol, obtaining a solution of OTA with a concentration of 1 mg OTA/mL (1000 µg/mL). This stock solution was diluted with methanol in order to obtain the appropriate work solutions (1, 10 and 100 mg/L). OTA solutions were stored in amber vials at 4 °C until the liquid chromatography coupled a fluorescence detector (HPLC-FLD) analysis. Acetonitrile, methanol, water, ethyl acetate (all of HPLC grade) and acetic acid were purchased from Merck (Whitehouse Station, Hunterdon, NJ, USA).

### 5.2. Yeast Strains and Culture Conditions

Two strains of *Saccharomyces cerevisiae* were investigated in this study. The first is a commercial strain (Levulin FB, OenoFrance, Magenta, France) commonly used in the winemaking process. The yeast was rehydrated abiding by the manufacturer’s instructions. Afterwards, a surface seeding of about 200 µL of the mixture obtained on YPD agar medium was performed. Subsequently, the Petri dishes were incubated at 28 °C for 3 days. The second *S. cerevisiae,* called SC5, was isolated from table grapes (*Vitis vinifera* L.) and purified on Petri dishes containing YPD agar medium. The SC5 strain was determined through a sequence analysis of the whole ITS region, including ITS1, ITS2 and the intervening 5,8S rRNA gene. The primers ITS1 and ITS4 were used as reported by White et al. [40]. Sequence analyses of the purified DNAs were conducted using a BigDye Terminator cycle sequencing kit V3.1 (Applied Biosystems, Foster City, CA, USA) and an Applied Biosystems 3130XL Capillary DNA Sequencer machine (Foster City, CA, USA). A sequence similarity was recorded through a BLAST analysis [41] using the GenBank DNA databases (http://www.ncbi.nlm.nih.gov/; 5 January 2022). The sequence was deposited in the GenBank database under the accession number OM080265.

### 5.3. Preparation of Cell Yeasts

In order to prepare dead and viable cells of yeasts so as to conduct assays, growing colonies of both yeasts were harvested from culture in YPD agar medium plates and were inoculated to 100 mL of YPD broth (1% yeast extract, 2% bacteriological peptone, 2% dextrose) and incubated for 48 h at 28 °C with rotary shaking (120 rpm).

After incubation, cells were harvested by centrifugation at 5000× *g* for 10 min at 4 °C and were washed three times with saline phosphate buffer (PBS; pH = 7.2). The resulting yeast pellets presented the viable cells. To prepare dead cells, half of the obtained yeast pellet was suspended in 100 mL PBS and kept heated to 80 °C for 1 h. Subsequently, the heat-treated cell suspension was cooled and centrifuged at 5000× *g* for 10 min at 20 °C. Afterwards, dead cells were collected and washed twice with PBS [26].

### 5.4. A. Carbonarius Strain and Preparation of Spore Suspension

Various strains of *A. carbonarius* which were isolated from table grapes (Italia Muscat cultivar) (*Vitis vinifera* L.) and gathered at a mature stage from the Zaghouan region (northeastern part of Tunisia), proved to be OTA producers on CYA medium [10]. CYA medium was prepared by dissolving 30 g of saccharose, 5 g of yeast extract, 50 mL of solution A (obtained by dissolving 40 g of NaNO_3_, 10 g of KCl, 10 g of MgSO_4_·7H_2_O and 0.2 g of FeSO_4_·7H_2_O in 1 L of distilled water), 50 mL of solution B (obtained by dissolving 20 g of K_2_HPO_4_ in 1 L of distilled water), 1 mL of metallic solution (solution C: prepared by dissolving 10 g of ZnSO_4_·7H_2_O, 5 g of CuSO_4_,H_2_O in 1 L of distilled water) and 15 g of Bacto agar in 1 L of distilled water with pH adjusted to 6.7.

The identification of these strains was performed microscopically, relying on the morphology of spores and conidial heads [42,43]. *A. carbonarius* strain AC36—a high-efficiency OTA producer (555.56 ng/g CYA)—was selected for this study. To confirm the morphological identification of the selected strain, a molecular characterization was carried out. This characterization was based on the amplification of internal transcribed spacer (ITS1-5.8S-ITS2) of the ribosomal DNA (rDNA) via the polymerase chain reaction (PCR), sequencing of the amplicon and analysis of similarity between the obtained sequences and those already deposited in the Center for Biotechnology and Information (NCBI, Rockville Pike Bethesda, Maryland, USA). The accession sequence of strain AC36 is OM182845.

A spore suspension of *A. carbonarius* strain was prepared from colonies, previously grown on potato dextrose agar (PDA) for seven days at 25 °C, in sterile distilled water involving Tween 80 (0.01%). It was adjusted to a concentration of 10^2^ spores/mL using a hemocytometer and was then kept at 4 °C.

### 5.5. Grape Juices Preparation

To explore OTA removal by dead and viable cells of *S. cerevisiae* strains from grape juice, two media were tested: (i) synthetic grape juice (SGM medium) with the following composition: 70 g of glucose, 30 g of fructose, 7 g/L of tartaric acid, 10 g of malic acid, 0.67 g of (NH_4_)_2_HPO_4_, 0.67 g of (NH_4_)_2_SO_4_, 1.5 g of KH_2_PO_4_, 0.75 g of MgSO_4_·7H_2_O, 0.15 g of NaCl, 0.15 g of CaCl_2_, 0.0015 g of CuCl_2_, 0.021 g of FeSO_4_.7H_2_O, 0.0075 g of ZnSO_4_, 0.05 g of catechin and 1 L distilled water. The pH of the medium was adjusted to 4.2 with KOH (2N). This juice was artificially contaminated with OTA (10 µg/L). (ii) Natural contaminated red grape juice extracted from table grapes (red globe variety) was contaminated with 6.64 µg/L of OTA, by coating berries for 20 min with the spore suspension of *A. carbonarius* (AC36). The coated grapes were incubated for 6 days at 30 °C, then berries were homogenized using a hand blender. In order to remove spores of *A. carbonarius* (AC36), the obtained juice was centrifuged at 5000× *g* for 10 min at 4 °C, and the supernatant was gathered and filtered through a 0.45 µm syringe-driven filter unit (MillexR SLHV 013 N K, Millipore, Bedford, MA, USA).

### 5.6. Evaluation of OTA Reduction from Grape Juice

To evaluate the ability of living and heat-treated cells of *S. cerevisiae* strains to remove ochratoxin from synthetic and natural contaminated grape juices, cells were inoculated in each medium with a concentration of 20 g/L, corresponding to 10^7^ cells/mL, determined using a hemocytometer. Subsequently, suspensions were incubated for 60 min at 30 °C with rotary shaking (400 rpm) [26]. After incubation, yeast cells and supernatant were separated by centrifugation at 6000× *g* for 20 min at 4 °C, and 1 mL of supernatant from each sample underwent OTA extraction and purification before HPLC analysis. A control treatment consisting of yeast-free natural and synthetic contaminated grape juices was included in the experiment and all assays were performed in triplicate

### 5.7. OTA Extraction and HPLC Analysis

OTA was extracted from supernatant samples and purified according to the method adopted by Bejaoui et al. [26]. For all samples, 1 mL of supernatant was extracted twice with ethyl acetate (*v*/*v*) using an Ultraturax (D-160 Homogenizer, Scilogex, Berlin, CT, USA) into a 2 mL Eppendorf Tube^TM^. The upper phase of each Eppendorf Tube^TM^ corresponding to the obtained extract was then evaporated using Speedvac (Thermo Fischer Scientific, Waltham, MA, USA) until it became dry. Subsequently, it was dissolved in 0.5 mL of methanol (HPLC grade) in order to solubilize the OTA. After that, methanolic extracts were injected into a HPLC system that was equipped with a C18 column (Waters Spherisorb 5 µm, ODS2, 4.6 × 250 mm, Milford, MA, USA). OTA detection was achieved with a fluorescence detector (Waters 474, Milford, MA, USA) at λexc 330 nm and λem 460 nm. The mobile phase involved acetonitrile-water-acetic acid (57:41:2) (1.0 mL/mn). Detection and quantification limits (LOD and LOQ) were 0.3 ng OTA/mL and 0.5 ng OTA/mL, respectively. The percentage of OTA reduction under each type of yeast was compared to the control.

### 5.8. Statistical Analysis

All experiments were carried out in triplicate, and the data were presented as means ± standard deviation (SD). All data were evaluated statistically through analysis of variance (ANOVA) using the Statistica software (version 5.0, StatSoft, Inc., Tulsa, OK, USA). Duncan’s multiple range tests were conducted to assess the differences among the factor levels studied at 5% significance level.

## Figures and Tables

**Table 1:** OTA concentrations in synthetic grape juice after inoculation with yeast cells.

*Saccharomyces cerevisiae* Strains	Concentration of OTA (µg/L) (±SD)	
	Viable Cells	Heat-Treated Cells
Control	9.81 ± 0.12 ^a^	9.81 ± 0.12 ^a^
Levulin FB	7.11 ± 0.06 ^b^	3.63 ± 0.21 ^e^
SC5	6.38 ± 0.23 ^c^	5.41 ± 0.19 ^d^

Data in the table are means ± SD of triplicate assays. ^a,b,c,d,e^ Values with the same superscript are not significantly different (*p* < 0.05) according to Duncan test.

**Table 2:** OTA concentrations in natural grape juice after inoculation with yeast cells.

*Saccharomyces cerevisiae* Strains	Concentration of OTA (µg/L) (±SD)	
	Viable Cells	Heat-Treated Cells
Control	6.64 ± 0.19 ^a^	6.64 ± 0.19 ^a^
Levulin FB	5.21 ± 0.11 ^b^	3.36 ± 0.09 ^d^
SC5	4.46 ± 0.02 ^b^	4.02 ± 0.03 ^c^

Data in the table are means ± SD of triplicate assays. ^a,b,c,d^ Values with the same superscript are not significantly different (*p* < 0.05) according to Duncan test.

## Data Availability

Not applicable.
